# Yeast Two Hybrid Analyses Reveal Novel Binary Interactions between Human Cytomegalovirus-Encoded Virion Proteins

**DOI:** 10.1371/journal.pone.0017796

**Published:** 2011-04-01

**Authors:** Aaron To, Yong Bai, Ao Shen, Hao Gong, Sean Umamoto, Sangwei Lu, Fenyong Liu

**Affiliations:** 1 Division of Infectious Diseases and Vaccinology, School of Public Health, University of California, Berkeley, California, United States of America; 2 Program in Comparative Biochemistry, University of California, Berkeley, California, United States of America; University of Nebraska – Lincoln, United States of America

## Abstract

Human cytomegalovirus (HCMV) is the largest human herpesvirus and its virion contains many viral encoded proteins found in the capsid, tegument, and envelope. In this study, we carried out a yeast two-hybrid (YTH) analysis to study potential binary interactions among 56 HCMV-encoded virion proteins. We have tested more than 3,500 pairwise combinations for binary interactions in the YTH analysis, and identified 79 potential interactions that involve 37 proteins. Forty five of the 79 interactions were also identified in human cells expressing the viral proteins by co-immunoprecipitation (co-IP) experiments. To our knowledge, 58 of the 79 interactions revealed by YTH analysis, including those 24 that were also identified in co-IP experiments, have not been reported before. Novel potential interactions were found between viral capsid proteins and tegument proteins, between tegument proteins, between tegument proteins and envelope proteins, and between envelope proteins. Furthermore, both the YTH and co-IP experiments have identified 9, 7, and 5 interactions that were involved with UL25, UL24, and UL89, respectively, suggesting that these “hub” proteins may function as the organizing centers for connecting multiple virion proteins in the mature virion and for recruiting other virion proteins during virion maturation and assembly. Our study provides a framework to study potential interactions between HCMV proteins and investigate the roles of protein-protein interactions in HCMV virion formation or maturation process.

## Introduction

Human cytomegalovirus (HCMV) is a member of the human herpesvirus family, which includes herpes simplex virus 1 (HSV-1), Varicella-zoster virus (VZV), Epstein-Barr virus (EBV), and Kaposi's sarcoma-associated herpesvirus (KSHV) [1]. This virus is an important opportunistic pathogen affecting individuals whose immune functions are compromised or immature [1], [2]. For example, HCMV is a leading cause of retinitis-associated blindness and other debilitating conditions such as pneumonia and enteritis among AIDS patients [3], [4]. Moreover, this virus causes mental and behavioral dysfunctions in children that have been infected *in utero* [1], [2]. Understanding the mechanism of how HCMV replicates and how viral proteins interact is critical in developing new compounds and novel strategies to control HCMV infections and prevent HCMV-associated diseases.

HCMV is the largest human herpesvirus, which encodes more than 150 open reading frames (ORFs) [5], [6], [7]. Its virion structure is also the largest and most complicated among human herpesviruses [8], [9]. Like other herpesviruses, HCMV virion is composed of an icosahedral capsid that contains a linear double-stranded DNA genome with attached proteins and an outer layer of proteins termed the tegument, surrounded by a viral envelope, which is derived from the cellular lipid bilayer and contains viral envelope glycoproteins [10], [11]. The capsid, which is exclusively assembled in the nucleus, contains five viral proteins encoded by open reading frame (ORF) UL86 (major capsid protein (MCP)), UL85 (minor capsid protein), UL80 (capsid assembly protein), UL48.5 (small capsid protein), and UL46 (minor capsid protein binding protein) [10], [11]. UL86 is the most abundant capsid protein and, together with UL85, UL48.5, and UL46, is present on the surface. The capsid is surrounded by the tegument, which is acquired in both the nucleus and cytoplasm of the infected cells. Mass spectrometry analysis of HCMV infectious particles indicated that more than 30 HCMV proteins of at least 100 amino acids, including UL24, UL25, UL26, UL32, UL43, UL47, UL48, UL82, UL83, UL94, UL99, US22, US23, US24, UL44, UL45, UL54, UL57, UL69, UL72, UL84, UL89, UL97, UL122, UL35, UL51, UL71, UL79, UL88, UL96, UL103, UL104, and UL112, were found in the tegument compartment [12], [13]. Quantitative proteomic analysis of HCMV virions suggested that the amounts of these proteins varied, with UL83 and UL99 as the most and least abundant tegument proteins, accounting for 15.4% and less than 0.1% of the total virion mass, respectively [12].

During virion maturation, nucleocapsids bud through nuclear membranes by acquiring a part of tegument and envelope. Subsequently, the cytoplasmic viral capsids containing tegument are enveloped further by budding into the trans-Golgi network or a closely apposed cellular compartment. More than 15 virally encoded envelope proteins, including RL10, UL5, UL22A (also called UL21.5), UL33, UL38, UL41A, UL50, UL55, UL73, UL74, UL75, UL77, UL93, UL100, UL115, UL119, UL132, and US27, have been found in the virion [12], [13].

The structure of the HCMV capsid has been studied by cryo-electron microscopy (cryo-EM) [8], [9] and recently refined to a resolution of 12.5 A [14]. In addition, interactions between capsid proteins have been investigated by yeast (*Saccharomyces cerevisiae*) two-hybrid (YTH) analysis as well as numerous biochemical and genetic approaches [14], [15], [16], [17], [18], [19], [20]. In contrast, the structure of the HCMV tegument is largely unknown. By cryo-EM, an icosahedrally ordered tegument density was visualized in HCMV particles when compared to precursor capsids prior to DNA encapsidation [8], [9]. However, the identity of the protein components of the tegument density has not been clearly determined. Moreover, the orientation and interaction of HCMV proteins in the virion have not been extensively studied.

Much of what is currently known about the interactions among herpesvirus capsid and tegument proteins come from various studies involving protein assays and in particular, YTH analyses [20], [21], [22], [23], [24]. Large-scale YTH analyses [25] have also been applied to interactome studies of many organisms such as *Homo sapiens*, *Drosophila melanogaster*, *Caenorhabditis elegans*, *Saccharomyces cerevisiae*, *Plasmodium falciparum*, and *Helicobacter pylori* [26], [27], [28], [29], [30]. In complex organisms where their genome sizes are relatively large, it is difficult to assess the importance of each individual protein to the systems. Therefore, it is important to map the global interactome to assess the true significance of each protein. Global genetic YTH analysis was also used to study the interactions between proteins encoded by vaccinia virus [31] and five herpesviruses, which include herpes simplex virus 1 (HSV-1), Varicella-zoster virus (VZV), Epstein-Barr virus (EBV), murine cytomegalovirus (MCMV), and Kaposi's sarcoma-associated herpesvirus (KSHV) [21], [22], [23], [24]. Furthermore, the potential interactions among 5 capsid proteins and 28 tegument proteins of HCMV have recently been investigated using the YTH approach [20]. These results have provided significant insights into the interactions among proteins encoded by herpesviruses.

In this study, we have carried out a comprehensive YTH analysis to identify potential interactions among 56 HCMV virion proteins, which include 5 capsid proteins, 33 tegument proteins, and 18 envelope proteins. We have identified 79 pairs of potential interactions that are involved in viral capsid proteins, tegument proteins, and envelope proteins. Of the 79 interactions, 58 have not been previously identified to the best of our knowledge, while 21 of them have been reported. Forty-five of these 79 putative interactions were also identified in human cells by co-immunoprecipitation (co-IP) experiments. Our results indicate the presence of several HCMV proteins that serve as “hubs” for interactions with numerous protein partners, thereby may function as an organizing center for connecting viral proteins in the mature virion and for recruiting other virion proteins. The interactions identified in this study provide a framework to study potential interactions between HCMV proteins and to investigate the functional roles of protein-protein interactions in HCMV virion assembly.

## Results

### Cloning of HCMV ORFs for yeast two-hybrid analysis

We have previously determined the genomic sequence of a HCMV Towne strain that was cloned into a bacterial artificial chromosome (BAC) vector [32] and was maintained as a single copy BAC-based plasmid in *E.coli* [7]. When introduced into human cells, the viral DNA sequence of this construct, Towne_BAC_, produces infectious progeny and retains wild type growth characteristic *in vitro*. These results suggested that the genome sequence of Towne_BAC_ encodes a collection of functional viral genes responsible for viral replication and infection [7], [33].

We then used the locally written Unix-based scripts or automation of GCG package to analyze the obtained Towne_BAC_ sequence and determine the coding sequences for ORFs that are 100 codons or longer. Each ORF was compared with the set of ORFs that had been predicted or found in all the HCMV strains for which sequences have been determined [5], [6], [7], [33], [34], [35]. This analysis suggested that the Towne_BAC_ sequence encodes at least 150 ORFs with 100 amino acids or longer, and that all these ORFs align with those found in other HCMV strains [7] (data not shown).

To study potential interactions among HCMV-encoded proteins in viral infectious particles, we cloned and expressed, in the yeast-two hybrid system, 56 HCMV ORFs that encode proteins that have been identified as virion components by mass spectrometry [12], [13]. Those cloned and expressed ORFs include 5 capsid proteins (UL46, UL48.5, UL80, UL85, and UL86), 33 tegument proteins (UL24, UL25, UL26, UL32, UL43, UL47, UL48 cloned individually as N′ and C′-terminal truncated fragments, UL82, UL83, UL94, UL99, US22, US23, US24, UL44, UL45, UL54, UL57, UL69, UL72, UL84, UL89 exon 1 (UL89.1)/UL89 exon 2 (UL89.2), UL97, UL122, UL35, UL51, UL71, UL79, UL88, UL96, UL103, UL104, and UL112 exon 1 (UL112.1)/exon 2 (UL112.2)), and 18 envelope proteins (RL10, UL5, UL22A (also called UL21.5), UL33, UL38, UL41A, UL50, UL55, UL73, UL74, UL75, UL77, UL93, UL100, UL115, UL119, UL132, US27).

We initially selected an optimal PCR primer pair for each ORF (Supporting Information Table S1). The primer pairs used for amplification of the viral sequences were constructed as follows. The forward primer contained (from 5′ to 3′) the sequence immediately after the predicted translation initiation codon and 20–25 additional nucleotides of coding sequence. The reverse primer contained the reverse complement of both the predicted translation termination codon and the preceding 20–25 nucleotides at the end of the ORF (Supporting Information Table S1). In addition, these primers also contain 15–20 nucleotide common sequences that contain sites for restriction enzymes for cloning of the PCR products into the YTH screen vectors (pGADT7 and pGBKT7) and the mammalian expression vectors (pCMV-HA and pCMV-Myc) (Supporting Information Table S1).

**Figure 1:**
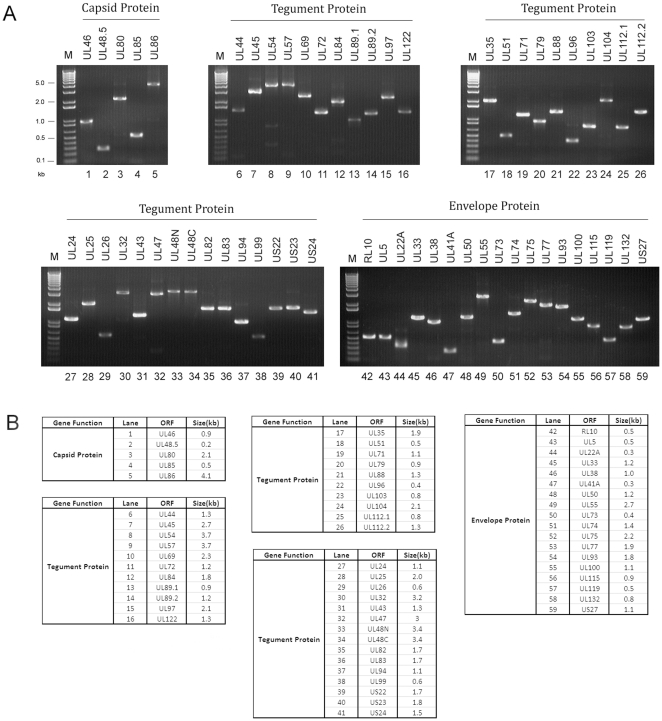
HCMV ORF sequences generated by PCR and cloned in yeast and mammalian expression vectors. (A) The PCR products for the HCMV ORFs separated on 1% agarose gels. (B) The predicted sizes of the PCR products for the HCMV ORFs based on the PCR primers used.

Each ORF encoding HCMV virion proteins was amplified individually by PCR. The amplified PCR products covered the entire ORFs minus the translation initiation codon (Figure 1), and were cloned into both the yeast two-hybrid screen “prey” pGADT7 and “bait” pGBKT7 vectors. We generated a collection of 118 constructs that contained the sequences of the 56 HCMV ORFs, including those coding for exons 1 and 2 of UL89, the amino and carboxyl domains of UL48, and exons 1 and 2 of UL112 (Figure 1, Supporting Information Table S2). The viral ORF sequences in these constructs were confirmed using DNA sequencing analysis (data not shown).

### Identification of potential interactions among HCMV-encoded virion proteins using YTH analysis

**Figure 2:**
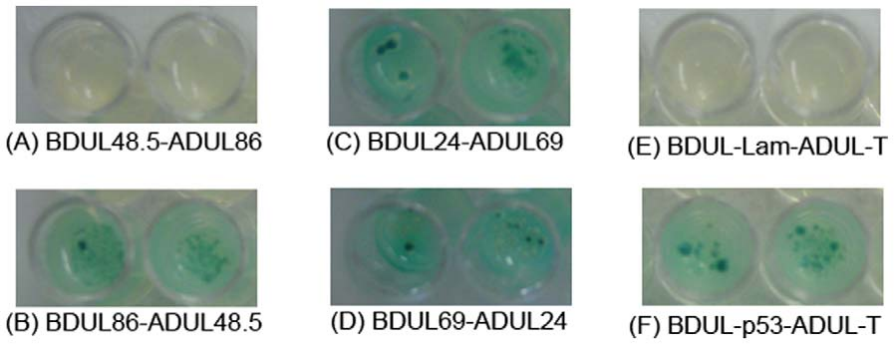
Protein-protein interactions between HCMV ORFs as identified by YTH analysis. In (A–B), a directional interaction is shown with the interaction existing only in BDUL86-ADUL48.5 (B) but not in BDUL48.5-ADUL86 (A). Interactions detected only in one direction are common in YTH assays and are most likely due to steric hindrance of either bait or prey fusion proteins [21], [22], [23]. In (C–D), an example of reciprocal interactions is shown with BDUL24-ADUL69 and the reverse combination. In (E–F), BDUL-Lam and BDUL-p53 are the human lamin C and murine p53 proteins fused to the BD domain of GAL4, respectively, while ADUL-T is the SV40 large T-antigen fused to the AD domain of GAL4. Murine p53 and SV40 T-antigen interact and serve as a positive control (F) while human lamin C and SV40 T-antigen do not interact and serve as a negative control (E). Diploids were plated on SD/-Leu/-Trp/-Ade/-His/X-α-Gal agar plates to test for protein-protein interactions. Positive interactions had blue yeast colony growth (B, C, D, and F), whereas no interactions were shown by the absence of growth (A and E). We observed similar growth of the diploid yeast cells representing the positive interactions.

Our strategy was to test each pair-wise combination of HCMV-encoded virion proteins in the YTH assay using a 48-well protein array format. The array consisted of a set of yeast transformants, each expressing a HCMV ORF as a hybrid protein with the Gal4 DNA binding domain (BD). This array was mated to yeast transformants of the opposite mating type carrying one of the HCMV ORFs as a hybrid protein with the Gal4 activation domain (AD). The resulting diploids were cultured on the medium selective for the two-hybrid reporter genes. In this approach, a single protein could be tested for binary interaction with the protein encoded by every cloned HCMV ORF in the array. By generating a different set of activation and DNA binding domain hybrids with HCMV virion proteins, we were able to carry out the mating and selection experiments to assay all of the ∼3500 combinations (Figure 2, Supporting Information Table S2).

To generate the yeast transformants for YTH analysis, DNAs from each of the 59 generated “bait” pGBKT7-derived constructs were isolated from *E.coli*, and transformed into yeast strain AH109, resulting in a collection of different yeast transformants that expressed a set of 59 DNA-binding domain (BD) hybrid proteins (Supporting Information Table S2). Similarly, DNAs from the generated “prey” pGADT7-derived constructs were isolated and transformed into yeast strain Y187 to generate yeast transformants expressing a set of 59 activation domain (AD) hybrid proteins.

Prior to performing mating experiments, all 59 pGBKT7-fusion proteins in AH109 strains were tested for autoactivation. Seven AH109 strains, which contained ORFs UL48.5, UL26, UL48N, UL48C, UL94, US23, and UL51, were determined to be autoactivators in the absence of any pGADT7-cloned ORFs (Table 1), and subsequently eliminated from any further mating experiments. Each of the remaining 52 AH109 strains was allowed to mate with each of the 59 constructed Y187 strains in duplicate, and the diploid cells were plated in duplicate on selective medium. Besides selecting for the presence of the two plasmids based on leucine and tryptophan selection, we employed a high stringency selection of positive protein interactions by using 3 more reporter genes to eliminate potential false positives: histidine, adenine, and Mel1, which encodes alpha-galactosidase for blue/white screening. The growth of colonies of the diploid yeast cells with a blue staining within 3 weeks after plating was scored as a positive pair-wise interaction between the two proteins tested while other results (e.g. no growth of yeast colonies) were scored as a negative interaction (Figure 2, Supporting Information Table S2). There was no significant difference in the growth of the diploid yeast cells representing all the positive interactions (data not shown).

**Table 1:** Protein-protein interactions of HCMV virion proteins that have either been previously reported or suggested, and were positive in our YTH and co-IP analysis.

BD ORFs and Function		AD ORFs and Function		Co-IP	Reported in HCMV	Reported in other Herpesviruses	References
**Capsid Protein-Capsid Protein Interactions**							
UL46	Minor capsid protein binding protein (E)	UL85	Minor capsid protein (E)	+	UL46-UL85	UL38-UL18 (HSV-1)	[10], [41]
UL80	Protease/Capsid assembly precursor (E)	UL80	Protease/Capsid assembly precursor (E)	+	UL80.5-UL80.5	UL26.5-UL26.5 (HSV-1); Orf33-Orf33 (VZV)	[17], [18], [22], [41]
UL85	Minor capsid protein (E)	UL85	Minor capsid protein (E)	+	UL85-UL85		[15], [16]
UL86	Major capsid protein (E)	UL48.5	Smallest capsid protein (E)	+	UL86-UL48/49		[19]
**Tegument Protein-Tegument Protein Interactions**							
UL25	Tegument protein (NE)	UL25	Tegument protein (NE)	+	UL25-UL25		[20]
UL25	Tegument protein (NE)	UL26	Transcription (D)	+	UL25-UL26		[20]
UL32	Basic phosphoprotein (E)	UL35	Tegument (Transcription) (D)	+	UL32-UL35		[20]
UL32*	Basic phosphoprotein (E)	UL82	Transcription (D)	+	UL32-UL82		[20]
UL35*	Transcription (D)	UL82	Transcription (D)	+	UL35-UL82		[45]
UL43*	Tegument protein (NE)	UL83	Tegument (immunomodulation) (NE)	+	UL43-UL83		[20]
UL45	Ribonucleotide reductase homologue (NE)	UL25	Tegument protein (NE)	+	UL45-UL25		[20]
UL45	Ribonucleotide reductase homologue (NE)	UL69	Transcription (D)	+	UL45-UL69	Orf61-Orf57 (KSHV)	[22]
UL47	Tegument protein (D)	UL48N	Tegument protein (E)	+		UL37-UL36 (PRV), Orf63-Orf64 (KSHV)	[22], [24]
UL69	Transcription (D)	UL69	Transcription (D)	+	UL69-UL69	Orf57-Orf57 (KSHV)	[22], [36]
UL72	Unknown function (D)	UL89.2	DNA packaging/cleavage (E)	+		Orf54-Orf29b (KSHV)	[22], [24]
UL82	Transcription (D)	UL82	Transcription (D)	+		Orf16-Orf16 (VZV)	[22], [24]
UL82	Transcription (D)	UL94	Tegument protein (E)	+	UL82-UL94		[20]
UL88	Unknown function (D)	UL48N	Tegument protein (E)	+	UL88-UL48		[20]
UL99	Envelopment (E)	UL94	Tegument Protein (E)	+	UL99-UL94	UL11-UL16 (HSV-1)	[38]
UL112.1	Early protein (D)	UL112.1	Early protein (D)	+	UL112-UL112		[39]
**Envelope Protein-Envelope Protein Interactions**							
UL77	DNA packaging/cleavage (E)	UL77	DNA packaging/cleavage (E)	+		Orf34-Orf34 (VZV)	[22], [24]

Interactions marked by an asterisk represent reciprocal interactions. “+” represents positive interactions identified by co-IP experiments in human cells (co-IP). The ORFs in which deletion results in no viral growth, wildtype-like growth, and significant growth defect in foreskin fibroblasts are marked as “E” (essential), “NE” (non-essential), and “D” (defect) [7].

Two-hybrid screens may generate a significant number of false positives that represent random generation of histidine-positive colonies. This is possibly due to rearrangement and deletion of the DNA-binding domain plasmids, recombinational events between the DNA-binding and activation domain plasmids, or genomic rearrangement of the host strain. To exclude these possibilities, three sets of experiments were carried out. First, plasmid DNAs from the yeast transformants were recovered and analyzed. Our results indicated that these constructs were present in all transformants examined and exhibited similar restriction enzyme profiles as those in *E. coli*. Second, all screens were carried out in duplicate to determine whether the results were reproducible. Third, any protein that resulted in histidine-positive growth with the empty vector controls (i.e. pGADT7 and pGBKT7) was classified as a false positive.

**Figure 3:**
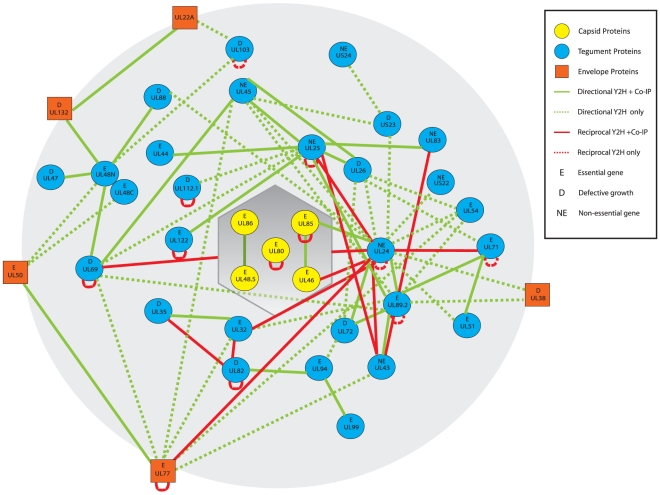
Interaction map of potential interactions between HCMV virion proteins as revealed by YTH analysis. The shaded hexagon represents the capsid and the shaded sphere represents the viral particle. The nodes (in yellow) within the capsid represent capsid proteins. The circular nodes in blue represent tegument proteins. The rectangular nodes in orange represent viral envelope proteins. The green lines represent interactions identified in one direction only and the red lines represent reciprocal interactions. The solid lines represent the interactions that were positive in both the YTH analysis and co-IP experiments, while the dashed lines represent the putative interactions that were positive only in the YTH analysis. The ORFs in which deletion resulted in no viral growth, wildtype-like growth, and significant growth defect in human foreskin fibroblasts are marked as “E” (essential), “NE” (non-essential), and “D” [7].

The >3000 combination screen of the mating of 52 HCMV Gal4 DNA-binding and 59 HCMV Gal4 activation domain fusion proteins revealed 79 positive interactions among 37 virion proteins (Supporting Information Table S2 and Figure 3). We identified 4 interactions between capsid proteins, 2 interactions between capsid proteins and tegument proteins, 58 interactions between tegument proteins, 12 interactions between tegument proteins and envelope proteins, and 3 interactions between envelope proteins (Table 1 and 2). The 37 participating proteins include all 5 capsid proteins (UL46, UL48.5, UL80, UL85, and UL86), 27 tegument proteins (UL24, UL25, UL26, UL32, UL43, UL47, UL48N/UL48C, UL82, UL83, UL94, UL99, US22, US23, US24, UL44, UL45, UL54, UL69, UL72, UL89.2, UL122, UL35, UL51, UL71, UL88, UL103, UL112.1), and 5 envelope proteins (UL22A, UL38, UL50, UL77, UL132). Of these 79 identified interactions, 58 are novel (Table 2) while the other 21 have previously been reported (Table 1).

**Table 2:** Putative protein-protein interactions of HCMV virion proteins that have not been previously reported and were determined by our YTH and co-IP analysis.

BD ORFs and Function		AD ORFs and Function		Co-IP
**Capsid Protein-Tegument Protein Interactions**				
UL24*	Tegument protein (NE)	UL46	Minor capsid protein binding protein (E)	+
UL24	Tegument protein (NE)	UL85	Minor capsid protein (E)	+
**Tegument Protein-Tegument Protein Interactions**				
UL24	Tegument protein (NE)	UL24	Tegument protein (NE)	− (putative)
UL24*	Tegument protein (NE)	UL25	Tegument protein (NE)	+
UL24	Tegument protein (NE)	UL26	Transcription (D)	− (putative)
UL24*	Tegument protein (NE)	UL32	Basic phosphoprotein (E)	+
UL24*	Tegument protein (NE)	UL43	Tegument protein (NE)	+
UL24	Tegument protein (NE)	UL51	DNA packaging/cleavage (E)	− (putative)
UL24*	Tegument protein (NE)	UL69	Transcription (D)	+
UL24*	Tegument protein (NE)	UL71	Unknown function (E)	+
UL24	Tegument protein (NE)	UL88	Unknown function (D)	− (putative)
UL24	Tegument protein (NE)	UL89.2	DNA packaging/cleavage (E)	+
UL24	Tegument protein (NE)	US22	Tegument protein (NE)	− (putative)
UL24	Tegument protein (NE)	US23	Tegument protein (D)	− (putative)
UL25*	Tegument protein (NE)	UL43	Tegument protein (NE)	+
UL25	Tegument protein (NE)	UL89.2	DNA packaging/cleavage (E)	+
UL32	Basic phosphoprotein (E)	UL89.2	DNA packaging/cleavage (E)	− (putative)
UL43	Tegument protein (NE)	UL89.2	DNA packaging/cleavage	+
UL44	DNA polymerase accessory protein (E)	UL25	Tegument protein (NE)	+
UL45	Ribonucleotide reductase homologue (NE)	UL24	Tegument protein (NE)	− (putative)
UL45	Ribonucleotide reductase homologue (NE)	UL26	Transcription (D)	+
UL45	Ribonucleotide reductase homologue (NE)	UL89.2	DNA packaging/cleavage (E)	− (putative)
UL45	Ribonucleotide reductase homologue (NE)	US23	Tegument protein (D)	− (putative)
UL54	DNA polymerase catalytic subunit (E)	UL24	Tegument protein (NE)	− (putative)
UL54	DNA polymerase catalytic subunit (E)	UL26	Transcription (D)	− (putative)
UL54	DNA polymerase catalytic subunit (E)	UL89.2	DNA packaging/cleavage (E)	− (putative)
UL69	Transcription (D)	UL25	Tegument protein (NE)	− (putative)
UL69	Transcription (D)	UL48N	Tegument protein (E)	+
UL69	Transcription (D)	UL89.2	DNA packaging/cleavage (E)	− (putative)
UL71	Unknown function (E)	UL26	Transcription (D)	− (putative)
UL71	Unknown function (E)	UL51	DNA packaging/cleavage (E)	+
UL71	Unknown function (E)	UL71	Unknown function (E)	− (putative)
UL71	Unknown function (E)	UL89.2	DNA packaging/cleavage (E)	+
UL72	Unknown function (D)	UL24	Tegument protein (NE)	+
UL72	Unknown function (D)	UL26	Transcription (D)	− (putative)
UL72	Unknown function (D)	UL94	Tegument Protein (E)	− (putative)
UL83	Tegument (immunomodulation) (NE)	UL25	Tegument protein (NE)	+
UL89.2	DNA packaging/cleavage (E)	UL89.2	DNA packaging/cleavage (E)	− (putative)
UL103	Unknown function (D)	UL48N	Tegument protein (E)	− (putative)
UL103	Unknown function (D)	UL103	Unknown function (D)	− (putative)
UL112.1	Early protein (D)	UL25	Tegument protein (NE)	− (putative)
UL122	IE-2 (Transcription) (E)	UL25	Tegument protein (NE)	+
UL122	IE-2 (Transcription) (E)	UL122	IE-2 (Transcription) (E)	+
US24	Tegument protein (NE)	US23	Tegument protein (D)	− (putative)
**Tegument Protein-Envelope Protein Interactions**				
UL24*	Tegument protein (NE)	UL77	DNA packaging/cleavage (E)	+
UL32	(Tegument) Basic phosphoprotein (E)	UL77	DNA packaging/cleavage (E)	− (putative)
UL38	Apoptosis Inhibitor (D)	UL24	Tegument protein (NE)	− (putative)
UL38	Apoptosis Inhibitor (D)	UL89.2	DNA packaging/cleavage (E)	− (putative)
UL43	Tegument protein (NE)	UL77	DNA packaging/cleavage (E)	− (putative)
UL45	Ribonucleotide reductase homologue (NE)	UL77	DNA packaging/cleavage (E)	− (putative)
UL50	Nuclear egress (E)	UL48N	Tegument protein (E)	− (putative)
UL50	Nuclear egress (E)	UL48C	Tegument protein (E)	− (putative)
UL54	DNA polymerase catalytic subunit (E)	UL77	DNA packaging/cleavage (E)	− (putative)
UL69	Transcription (D)	UL77	DNA packaging/cleavage (E)	− (putative)
UL103	Unknown function (D)	UL22A	Glycoprotein (D)	− (putative)
UL132	Glycoprotein (D)	UL48N	Tegument protein (E)	+
**Envelope Protein-Envelope Protein Interactions**				
UL50	Nuclear egress (E)	UL77	DNA packaging/cleavage (E)	+
UL132	Glycoprotein (D)	UL22A	Glycoprotein (D)	+

Interactions marked by an asterisk represent reciprocal interactions. “+” and “−” represent positive or negative interactions identified by co-IP experiments in human cells (co-IP). The ORFs in which deletion results in no viral growth, wildtype-like growth, and significant growth defect in foreskin fibroblasts are marked as “E” (essential), “NE” (non-essential), and “D” (defect) [7].

Twelve proteins, which include 2 capsid proteins (UL80 and UL85), 9 tegument proteins (UL24, UL25, UL82, UL69, UL89, UL122, UL71, UL103, and UL112), and 1 envelope protein (UL77), were found to be self-interacting. Previous analyses have shown the self-interaction of UL80, UL85, UL25, UL69, and UL112 [10], [15], [20], [36]. These results suggest that all 12 proteins may form dimers or self-assemble into polymers. These notions are consistent with previous observations that UL85 is the minor capsid protein that can self-assemble into pre-caspid particles and that UL80 self-interacts and forms a dimer (Table 1) [10], [15].

Of the 79 interactions identified, 11 of them were reciprocal interactions that were tested and confirmed in both orientations in the YTH assay (Supporting Information Table S2, Figures 2–3), strongly supporting the validity of these interactions. Excluding the 12 self-interactions and 11 reciprocal interactions, the remaining 56 interactions were detected in only one direction. Interactions detected only in one direction are common in YTH analysis and most likely are due to steric hindrance of either bait or prey fusion proteins [21], [22]. Among these 79 interactions, 18 were found to be involved with UL24 while 11 and 10 were with UL25 and UL89.2, respectively (Figure 3, Table 1 and 2). These results raise the possibility that these proteins may function as a hub or organizing center for connecting viral proteins in the mature virion and for recruiting other virion proteins during virion maturation and assembly. UL24, the only tegument protein identified in this study to interact with a capsid protein (e.g. UL46), may be especially important in this process.

### Expression of HCMV virion proteins and investigation of their interactions in human cells

**Figure 4:**
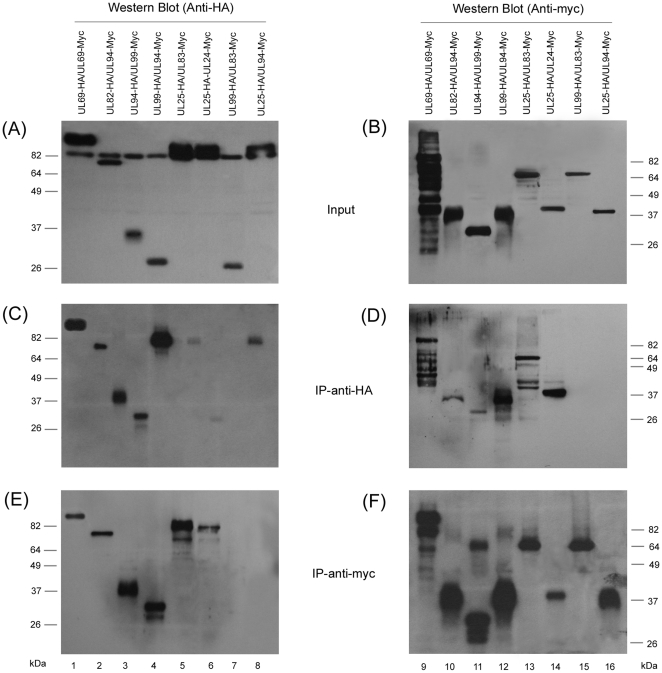
Co-immunoprecipitation (co-IP) of transiently expressed viral proteins. HeLa cells were co-transfected with a combination of two plasmids expressing HA- and myc-tagged proteins. Panels (A) and (B) are inputs that show the expression of HA- and myc-tagged proteins detected by Western blot analysis with anti-HA and anti-myc antibodies, respectively. Lanes 1 and 9 are from the same cell lysate, likewise, lanes 2 and 10, and so on. Lanes 7, 8, 15, and 16 are negative controls based on our YTH data that revealed no interactions. Panels (C) and (D) represent co-IP experiments (IP-anti-HA) with anti-HA agarose antibodies and visualized by Western blot analysis using anti-HA and anti-myc antibodies, respectively. Panels (E) and (F) are co-IP experiments (IP-anti-myc) with anti-myc agarose antibodies (IP-anti-Myc) and visualized by Western blot analysis using anti-HA and anti-myc antibodies, respectively. The protein species, which migrated at approximately 82 kDa and was detected by the anti-HA antibody in the input protein samples of the cell lysate (A) but not in the protein samples immunoprecipitated with either the anti-HA or anti-myc antibodies (C and E), may represent a cellular protein that non-specifically reacts with the anti-HA antibody.

A co-immunoprecipitation (co-IP) assay was employed to validate the yeast two-hybrid approach and to determine whether the identified interactions occur in human cells. Figure 4 shows the results of the co-IP experiments that examined six of the interactions identified in the YTH analysis, UL24-UL25, UL69-UL69, UL82-UL94, UL83-UL25, UL99-UL94 and its reciprocal, UL94-UL99 (lanes 1–6). Two pair interactions, UL99-UL83 and UL25-UL94, which showed no interactions in our YTH screen, were also included as control experiments (lanes 7 and 8). In these experiments, the HCMV ORFs were cloned into mammalian expression vectors pCMV-Myc and pCMV-HA, in which each ORF was expressed as either a fusion protein with a carboxyl terminal c-myc or HA epitope tag, respectively. HeLa and U373MG cells were transfected with the mammalian expression constructs and were harvested at 72 hours. To detect the expression of these proteins, transfected cells were lysed and proteins were separated electrophoretically on SDS-containing gels, transferred electrically to membranes, and reacted with anti-HA and anti-c-myc antibodies. UL24, UL25, UL69, UL82, UL83, UL94 and UL99 were detected as proteins of about 40, 80, 90, 70, 70, 40, and 25 kD, respectively (data not shown, Figure 4), consistent with their coding sequences of 358, 656, 743, 559, 561, 345, and 190 amino acids, as predicted from the HCMV Towne_BAC_ sequence [7]. The protein species, which migrated at approximately 82 kDa and was detected by the anti-HA antibody in the input protein samples of the cell lysate (Figure 4A) but not in the protein samples immunoprecipitated with either the anti-HA or anti-myc antibodies (Figure 4C and E), may represent a cellular protein that non-specifically reacts with the anti-HA antibody.

The six interactions revealed by our YTH analysis appeared to be identified in the co-IP experiments (Figure 4, lanes 1–6). As an example, to determine whether UL25 and UL94 interact with UL83 and UL99 respectively, protein lysates from the transfected cells were either first immunoprecipitated with anti-HA or anti-c-myc, and then immunoblotted with antibodies against the c-myc and HA epitope tags. The HA-tagged UL25 was co-precipitated with the myc-tagged UL83 (Figure 4C–F, lane 5 and 13), while the HA-tagged UL99 was found to be associated with the myc-tagged UL94 and vice versa (lane 3–4 and 11–12). In contrast, we observed, in control experiments, neither significant binding between the HA-tagged UL99 and the myc-tagged UL83 (lane 7) nor between the HA-tagged UL25 and the myc-tagged UL94 (lane 8). These results confirm the specificity of the co-IP assay and suggest that the UL25-UL83 and UL99-UL94 interactions may occur in human cells. These results are consistent with recent report of interactions between UL69-UL69, UL82-UL94, and UL99-UL94 revealed by YTH and co-IP analysis [20]. Similar co-IP experiments were carried out to study each of the 79 putative interactions identified in the YTH screens in human cells. Forty five of these interactions were positive in our experiments while 34 were negative (Table 1 and 2).

## Discussion

Large-scale YTH analyses have been applied to interactome studies of many organisms, including *Homo sapiens*, *Drosophila melanogaster*, *Caenorhabditis elegans*, *Saccharomyces cerevisiae*, *Plasmodium falciparum*, and *Helicobacter pylori* [26], [27], [28], [29], [30]. Global YTH analysis was also used to study the potential interactions between proteins encoded by vaccinia virus [31] and five herpesviruses HSV-1, MCMV, KSHV, VZV, and EBV [21], [22], [23]. Furthermore, the potential interactions among 5 capsid proteins and 28 tegument proteins of HCMV have recently been investigated using the YTH approach [20]. Although powerful in the amount of data generated by the many interactome studies, much work still remains to fully understand the biological significance of the identified interactions. Nonetheless, the interactome studies have proven to be very valuable starting points for predicting functions of unknown proteins when found to interact with known partners. In addition, they serve as building blocks for many systems-level studies [37]. In this study, we used the yeast two-hybrid screen approach to study potential binary interactions among HCMV-encoded virion proteins. Our YTH analysis revealed 79 potential interactions, 45 of which were also identified using co-IP experiments by expressing these proteins in human cells.

It has been reported that two-hybrid screens may generate significant number of false positives [25]. One type of false positives may represent random generation of histidine-positive colonies. This is possibly due to rearrangement and deletion of the DNA-binding domain plasmids, recombinational events between the DNA-binding and activation domain plasmids, and genomic rearrangement of the host strain. Other types of false positives may be due to either non-specific activation of different reporter systems or autoactivation, which is caused by activators of transcription with only the binding domain-fusion proteins. Similarly, false negatives may arise from the YTH screens. YTH systems that test for protein interactions in the nucleus are prone to false negatives due to the expression of proteins that are normally not found in the nuclear environment. Furthermore, this system may potentially miss interactions involving proteins with significant hydrophobic domains such as membrane proteins, which may not be folded correctly.

To exclude these possibilities, five different sets of experiments have been carried out. First, plasmid DNA from the yeast transformants was recovered and analyzed. Our results indicated that these constructs were present in all transformants examined and exhibited similar restriction enzyme profiles as those in *E. coli*, suggesting that there is no significant change or rearrangement of the sequence of the transformants during the screening process. Second, all screens were carried out in duplicate to determine whether the results were reproducible. Third, prior to any matings in our experiments, all of the binding domain-fusion proteins have been tested for autoactivation and those positive ones were subsequently removed from this study. Fourth, we have employed a high stringency selection in the YTH analysis by utilizing 4 different reporters to ensure the validity of the data. We also observed similar growth of the diploid yeast cells representing the positive interactions. Any protein that resulted in histidine-positive growth with the empty vector controls (i.e. pGADT7 and pGBKT7) was classified as a false positive. Fifth, many of the identified interactions were further examined by expressing these proteins in human cells. Whether they associate with each other in human cells was investigated by co-IP experiments.

To our best knowledge, 58 of these 79 interactions found in our YTH screens have not been reported previously while 17 have been found in HCMV and 4 in other herpesviruses (Table 1 and 2) [15], [16], [17], [20], [23], [24], [36], [38], [39], [40], [41]. Furthermore, all the previously identified 21 interactions and 24 of the new 58 interactions were positive in co-IP experiments, suggesting the presence of these interactions in human cells. The identified interactions can be classified into four different categories based on the locations of these viral proteins in the virion.

### (A) Interactions of viral capsid proteins

Extensive studies have been carried out to investigate the interactions between capsid proteins [10], [11]. Our YTH screens coupled with co-IP experiments revealed four interactions, all of which have been previously identified [15], [16], [17], [18], [19], [20], [42], validating our approach for identifying protein-protein interactions.

### (B) Interactions between viral capsid and tegument proteins

Both the YTH and co-IP analyses revealed novel potential interactions of tegument protein UL24 to capsid protein 46 and UL85 (Table 2), which have not been reported previously. It is believed that tegument proteins specifically interact with capsid proteins to initiate the formation of the tegument and to form mature infectious virions. By cryo-EM, an icosahedrally ordered tegument layer was visualized in HCMV particles when compared to precursor capsids prior to DNA encapsidation [8], [9]. Furthermore, distinct tegument densities were observed to interact with all of the structural components of the nucleocapsid. Possible functions of these capsid-proximal organized tegument proteins may be involved in serving as a nucleus for further tegument assembly. However, the identity of the tegument densities has not been clearly determined. So far only one interaction between HCMV tegument proteins and capsid proteins has been reported. Gibson and coworkers have shown that UL32 can bind to intranuclear HCMV capsids via its amino-third domain [43]. Previous studies using YTH screens as well as our results here failed to detect interactions of UL32 with any HCMV capsid proteins. This may possibly be because the region of the capsid protein that interacts with UL32 is only folded properly when the protein is present in the capsid, and therefore, the interactions between this protein and UL32 can not be detected by YTH screens as these proteins are expressed in the absence of capsid formation [43].

Our YTH and co-IP analyses provide the first direct evidence of potential interactions between tegument protein UL24 and capsid proteins UL46 and UL85 (Table 2 and Supporting Information Table S2, Figure 3). The UL24 protein has been detected in the tegument [12], [44]. Gene-deletion analyses indicate that UL24 is not essential for viral growth in human foreskin fibroblasts but is important for viral replication in microvascular endothelial cells [7]. However, its exact function is currently unknown. It is possible that UL24 may function as a “hub” to connect viral capsids to other tegument proteins that it interacts with, and facilitate tegument formation by recruiting other tegument proteins to the capsid.

### (C) Interactions among tegument proteins

It is generally believed that tegument proteins interact with each other as well as with human proteins to maintain the structure of the tegument and play an important role in recruiting proteins during tegument formation. Nineteen specific interactions among HCMV tegument proteins have been reported and include the self-interaction of UL25, UL44, UL45, UL69 and UL112, and the interaction of UL25-UL26, UL32-UL35, UL32-UL82, UL35-UL82, UL43-UL83, UL45-UL25, UL45-UL69, UL82-UL94, UL88-UL48, UL99-UL94, UL32-UL45, UL48-UL45, UL69-UL88, UL94-US22 [20], [22], [36], [39], [45].

Of the 79 interactions identified by our YTH analysis, 58 (>73%) are between tegument proteins (Table 1 and 2). Our results confirm the previously identified 13 interactions (i.e. self-interaction of UL25, UL69, and UL112; and interaction of UL25-UL26, UL32-UL35, UL32-UL82, UL35-UL82, UL43-UL83, UL45-UL25, UL45-UL69, UL82-UL94, UL88-UL48, and UL99-UL94) (Table 1). Furthermore, these results suggest the presence of 45 possible interactions between tegument proteins, 21 of which were also positive in co-IP experiments (Table 2). The salient features of these interactions are as follows:

1. Both YTH and co-IP experiments identified 7, 9, and 5 interactions that were involved with UL24, UL25, and UL89.2, respectively. Thus, UL24, UL25, and UL89 may function as a hub or organizing center for connecting multiple virion proteins in the mature virion and for recruiting other virion proteins during virion maturation and assembly.
2. Previous studies have suggested that proteins of similar functions may interact with each other to achieve their function synergistically and co-operatively [21], [22], [31]. For example, UL35 and UL82 have been shown to interact with each other, and form a complex that functions to activate viral transcription cooperatively and synergistically [45]. Identifying the binding partners of an unknown protein may shed light into its potential biological function. Excluding those interactions that are involved in UL24, UL25, and UL89.2, which may function to recruit tegument proteins, the majority (24) of the remaining 25 interactions identified in YTH experiments are either between essential genes or between genes that are important for viral replication in human foreskin fibroblasts (Table 1 and 2) [7]. For example, both the YTH and co-IP analysis identified the interactions of UL71, an essential protein, with UL51 and UL89, which are essential for viral DNA genome cleavage and packaging [1], [7], [46]. These results suggest that UL71 may play its essential role in supporting viral DNA encapsidation. Further studies will be carried out to investigate the roles of UL71 and its interactions with UL51 and UL89 in viral replication.

### (D) Interactions between viral tegument proteins and envelope proteins and among envelope proteins

Little is currently known about the interactions between viral tegument and envelope proteins. Our YTH analysis suggests 12 potential interactions between tegument proteins and envelope proteins. In particular, the UL24-UL77 and UL132-UL48 interactions were identified by both the YTH and co-IP analyses. Furthermore, novel interactions between envelope proteins, UL22A-UL132 and UL77-UL50, were also identified by both the YTH and co-IP experiments. Both UL77 and UL50 are essential for viral replication [7], [46]. However, the exact function of UL77 is currently unknown while UL50, along with a non-virion protein UL53, functions to dismantle the inner nuclear membrane and facilitate the budding of the nucleocapsid into the nuclear membrane during the initial step of acquiring the viral envelope [47], [48]. It will be interesting to investigate whether UL77 interacts with UL50 and exerts similar function in supporting the budding of the nucleocapsid through the nuclear membrane. UL132 encodes a glycoprotein that may play an important structural function during viral replication in vitro [49]. ORF UL22A, which is also called UL21.5, is believed to encode a RANTES decoy receptor glycoprotein found in the virion [12], [50]. It will be interesting to determine whether UL22A forms a complex with UL132 to exert their functions in supporting viral growth during HCMV infection.

We note that our results failed to detect a number of interactions between HCMV virion proteins that have been previously identified by YTH analysis, including the seven interactions (i.e. self-interaction of UL44 and UL45; interaction of UL32-UL45, UL48-UL45, UL69-UL88, UL94-US22, UL86-UL46) reported recently [20]. This may be due to the difference in the yeast strains, activation/DNA binding domain vectors, and the selection medium/reporters that were used for the studies. Little overlap of interactions identified by different research groups has been observed in the YTH studies of other systems including herpesviruses such as EBV and KSHV [21], [22], [23], [24]. There are also a number of previously reported interactions between viral envelope proteins that were not detected in our current study. This may not be surprising as most of the viral envelope proteins may not be folded properly in our YTH screen system, and therefore, may not possess the correct conformations for binding and interactions. Moreover, YTH systems that test for protein interactions in the nucleus are prone to false negatives due to the expression of proteins that are normally not found in the nuclear environment. These issues can be resolved by using affinity purification assays to detect interactions *in vivo*. However, co-IP experiments can only detect relatively stable, high affinity interactions while the YTH methods reveal transient, weak interactions. This is because the dissociation constant (K_d_) for a typical YTH assay is 10–100 µM compared to ∼10 mM for co-IP experiments [51]. Fifty-seven percent of the interactions revealed by our YTH analysis was confirmed in co-IP experiments, consistent with previous observations that about 50–60% of the YTH interactions are positive in immunoaffinity pulldown experiments in the studies of other viruses [21], [22], [23], [24], [31]. While we can not completely rule out the possibility that those interactions which are negative in co-IP experiments may not be present in mammalian cells, it is conceivable that these interactions are so transient and weak that they are only detected by the YTH approach but not by the immunoaffinity pulldown assay.

The HCMV virion represents one of the most complex viral particles found in nature. It contains more than 55 HCMV proteins of at least 100 amino acids, and in addition, at least 10 viral-encoded small peptides/proteins of less than 100 amino acids and over 70 human cellular proteins [12], [13]. Of the 56 ORFs we studied, 19 were either not found to interact between themselves or with any other of the 37 HCMV proteins. We can not completely exclude the possibility that there were interactions among themselves or with the other 37 ORFs in human cells that could not be detected by our YTH assays. It is also conceivable that these proteins may interact with the viral encoded small peptides, human proteins, and other constituents of the virion particles (e.g. lipids). Further studies to identify the partners of these proteins and study their potential interactions with the partners will provide insight into the mechanism of HCMV virion assembly and formation, and facilitate the development of novel compounds and new strategies for the treatment and prevention of HCMV infection.

## Materials and Methods

### Construction of plasmids

HCMV Towne_BAC_ (accession no. AY315197) [7] was used as the template for PCR amplification of HCMV ORFs . For genes that encode spliced transcripts, cDNAs from Towne_BAC_-infected cells were used as the templates. Table S1 (Supporting Information) lists the primers used for cloning HCMV sequences in the pGBKT7 bait and pGADT7 prey plasmids for expression in yeast and in pCMV-Myc and pCMV-HA for expressing in human cells (Clontech, Mountain View, CA). Each primer sequence contains an outer and inner restriction enzyme site for cloning the HCMV sequences at the multiple cloning site (MCS) of the yeast and mammalian expression plasmids, respectively (Table S1) (Supporting Information). PCR amplification was performed using iProof high-fidelity DNA polymerase (Bio-Rad, Inc., Hercules, CA). The resultant constructs were confirmed by restriction digest profile and sequencing.

### Yeast two-hybrid (YTH) analysis

The DNAs of the viral ORFs that were cloned into both pGBKT7 and pGADT7 were transformed into AH109 (*MAT* **a**) and Y187 (*MAT* **α**) yeast strains, respectively (Matchmaker 3 System, Clontech). AH109 strains harboring pGBKT7 plasmids were maintained in minimal SD media with tryptophan dropout supplement (SD/-Trp) while Y187 strains harboring pGADT7 plasmids were maintained in minimal SD media with leucine dropout supplement (SD/-Leu).

Prior to performing matings, individual AH109 strain was plated on SD/-Ade/-His/-Trp agar with 40 µg/ml X-α-Gal, and tested for autoactivation. AH109 strains containing the sequences of seven ORFs (UL48.5, UL26, UL48N, UL48C, UL94, US23, and UL51) were determined to be autoactivators in the absence of any pGADT7-cloned ORFs, and subsequently eliminated from further mating experiments.

Yeast mating was carried out by inoculating fresh colonies (<2 weeks old) of both AH109 and Y187 strains into 1.5 ml microcentrifuge tubes containing 0.5 ml of YPDA media and incubated at 30°C with shaking at 200 rpm for 24 hours. There were a total of 3068 possible combinations (52 AH109×59 Y187) and each mating combination was performed in duplicates. To select for diploids that have successfully been mated, yeast mating cultures were centrifuged at 14,000 rpm for 15 seconds, resuspended in 0.5 ml Tris-EDTA buffer, and plated in one well of 48-well plates containing 1 ml of SD/-Leu/-Trp agar and incubated at 30°C for 3–5 days. Diploid colonies from each successful mating were then picked from each well, resuspended with TE buffer, and plated in one well of 48-well plates containing 1 ml of SD/-Ade/-His/-Leu/-Trp (quadruple drop-out (QDO)) agar with 40 µg/ml of X-α-Gal.

Three weeks after plating the mated diploid yeasts, the QDO plates were scored for positive protein-protein interactions. QDO/X-α-Gal plates represent a high selection stringency for eliminating possible false positives. AH109 strains contain three reporters-ADE2, HIS3, and MEL1 (encodes α-galactosidase) under unique GAL4 upstream activating sequences (UASs) and TATA boxes. The mating between AH109 transformed with a plasmid expressing the GAL4 binding domain (BD)-p53 fusion protein and Y187 transformed with a plasmid expressing the GAL4 activation domain (AD)-T (SV40 T antigen) fusion protein was used as the positive control. The mating between AH109 transformed with a plasmid expressing the BD-Lamin fusion protein and Y187 transformed with a plasmid expressing AD-T was used as the negative control. Growth of the diploid yeast cells representing the positive interactions was further analyzed.

When scoring the duplicates, if both had no yeast growth after 3 weeks incubation, the fusion proteins were considered non-interacting partners. When the duplicates both had blue yeast colonies or if only one of the wells had blue yeast colonies, that mating combination was repeated until the results from two of three independent experiments are consistent.

### Co-immunoprecipitation (co-IP) analysis

HeLa and U373MG cells, which were obtained from American Type Culture Collection (ATCC) (Manassas, VA), were co-transfected with the DNAs of the generated expression constructs that were derived from pCMV-myc and pCMV-HA and contained the HCMV ORF sequences, with the aid of Lipofectamine 2000 (Invitrogen, Carlsbad, CA). In some experiments, U373MG cells expressing a T7 RNA polymerase, which supported HCMV lytic replication, were co-transfected with the constructs that were used for the YTH analysis. The expression of T7 polymerase in these cells induced the expression of the HCMV ORFs cloned in the pGBKT7 and pGADT7 vectors. Cell lysates were harvested at 3 days post-transfection. Co-immunoprecipitation experiments were performed using the ProFound Mammalian HA Tag IP/Co-IP and c-myc Tag IP/Co-IP kits following the manufacturer's protocol (Pierce, Rockford, IL).

### Western blot analysis

The denatured polypeptides from cell lysates were separated on SDS-polyacrylamide gels cross-linked with *N*,*N*″-methylenebisacrylamide, transferred electrically to nitrocellulose membranes [14], [52]. Detection of tagged proteins was performed by incubation with mouse anti-myc or rabbit anti-HA antibodies (Clontech, Mountain View, CA) and reacted in an enzyme-linked immunoassay with either anti-mouse IgG or anti-rabbit IgG conjugated with horse radish peroxidase (Vector Laboratories, Burlingame, CA) [53]. The membranes were subsequently stained with a chemiluminescent substrate with the aid of a Western chemiluminescent substrate kit.

## Supporting Information

Table S1 The primers used to clone the coding sequences of HCMV ORFs in yeast and mammalian expression vectors. The 5′ end of each primer contains two restriction enzyme sites for cloning into the expression plasmids.
(DOC)

Table S2 Yeast two-hybrid mating results between constructs that contained DNA sequences coding for 59 binding domain (BD) and 59 activation domain (AD) fusion proteins. “AA” represents BD fusion proteins that are identified as autoactivators. “−” represents matings that resulted in no interactions. Highlighted “P” represents positive interactions by YTH mating. There was no significant difference in the growth of the diploid yeast cells representing all the positive interactions.
(DOC)
