# Supplementary material for: Yeast Two Hybrid Analyses Reveal Novel Binary Interactions between Human Cytomegalovirus-Encoded Virion Proteins
Source: PLoS One. 2011 Apr 1;6(4):e17796. doi: 10.1371/journal.pone.0017796 (PMC3069980; doi:10.1371/journal.pone.0017796)
Supplement: Table S1 — The primers used to clone the coding sequences of HCMV ORFs in yeast and mammalian expression vectors. The 5′ end of each primer contains two restriction enzyme sites for cloning into the expression plasmids. (DOC) [file pone.0017796.s001.doc]

**SUPPORTING INFORMATION**

Table S1. The primers used to clone the coding sequences of HCMV ORFs in yeast and mammalian expression vectors. The 5’ end of each primer contains two restriction enzyme sites for cloning into the expression plasmids.

|  | **Upstream Primer** | **Downstream Primer** |
| --- | --- | --- |
| **UL5** | CCGGAATTCTTGTCGACCTTTCTAGGCTACTCTGACTGT | CGCGGATCCGGTACCCTACACGGTAGCGACGAGA |
| **UL22A** | GGGAATTCCATATGTTGTCGACCGCTCGGAGGCTATGGATACTGA | CCGGAATTCGGTACCCTACTGGGTCTTTTCATTTTCT |
| **UL24** | CCGGAATTCGAGATCTCTGAGGAGACCCGGGCGGGA | CGCGGATCCGGTACC TCAACGGTGCTGACGTCCTT |
| **UL25** | CCGGAATTCAAGGCCATGGAGGCCTCGTCGCGGCGTCGCAGCT | CGCGGATCCGGTACCTCAGCAACAGTATTCCCCGCT |
| **UL26** | CCGGAATTCTTGTCGACCACGAGCAGGCGCGCACCC | CGCGGATCCGGTACCTTACGGCAACAGCGCTGATGG |
| **UL32** | GGGAATTCCATATGAAGATCTCTAGTTTGCAGTTTATCGGTCTACA | CGCGGATCCGCGGCCGCCTATTCCTCCGTGTTCTTAATCTT |
| **UL33** | CCGGAATTCTTGTCGACCACGGAGACGCTATCCGCCAT | CGCGGATCCCTCGAGTCATACCCCGCTGAGGTTATGA |
| **UL35** | GGGAATTCCATATGTCTCGAGGTGCTCAAGGATCGCGAGCCCCA | CGCGGATCCGCGGCCGCTTAGAGATGCCGTAGATTTTCGG |
| **UL38** | GGGAATTCCATATGTTGTCGACCACTACGACCACGCATAGCACC | CCGGAATTCGGTACCCTAGACCACGACCACCATCTG |
| **UL41A** | CCGGAATTCTCTCGAGGTACACTCTTTTGCCGCACCG | CGCGGATCCGCGGCCGCTTAAAAGTCTGTGTCCGACTC |
| **UL43** | GGGAATTCCATATGTTGTCGACCGAGAAAACGCCGGCGGAGACG | CCGGAATTCGGTACCTCACCTTCGAGCAAAGAGCCCCT |
| **UL44** | GGGAATTCCATATGTTGTCGACCGATCGCAAGACGCGCCTCTC | CGCGGATCCGGTACCCTAGCCGCACTTTTGCTTCTTG |
| **UL45** | CCGGAATTCTTGTCGACCAATCCGGCTGACGCGGACGA | CGCGGATCCGCGGCCGCCTAAGAGGCACAGTACTTATATAC |
| **UL46** | GGGAATTCCATATGTCTCGAGGTGATGCACGCGCGGTGGCCAAA | CGCGGATCCGCGGCCGCTCAGACGAATTCTCGAAAGTCTCC |
| **UL47** | CCGGAATTCTTGTCGACCATGGCGAGGCGCACGGTAGATT | CGCGGATCCGGTACCTCATGGCGAGGCCGGCGGCA |
| **UL48N** | CCGGAATTCAAAGTCACGCAGGCCAGCTGC | CCGGAATTCTTACAGAAGTTGCGGACCCAAAATTTG |
| **UL48C** | CCGGAATTCATCCATGAGACGCAGCAGGCC | CCGGAATTCTTACAAAAGATAGAGAAACCGCATGT |
| **UL48.5** | CCGGAATTCTTGTCGACCTCTAACACCGCGCCGGGACC | CGCGGATCCGGTACCTCAGCGCCGGGTGCGCGAC |
| **UL50** | CCGGAATTCTCTCGAGGTGAGATGAACAAGGTTCTCCATCA | CGCGGATCCGCGGCCGCTCAGTCGCGGTGTGCGGAGC |
| **UL51** | CCGGAATTCTTGTCGACCTCCTGGGCTAAGCAGCGGGT | CGCGGATCCGGTACCTTATTTACCCGGCGCCGGCT |
| **UL54** | GGGAATTCCATATGTTTTTCAACCCGTATCTGAGCGG | CCGGAATTCTCAACAGCATTCGTGCGCCTTG |
| **UL55** | CGCGGATCCGGGCCATGGAGGCCGAATCCAGGATCTGGTGCCTG | CGCGGATCCGGTACCTCAGACGTTCTCTTCTTCGTCG |
| **UL57** | GGGAATTCCATATGGCGGCCGCTAGCCACGAGGAACTAACCGC | CGCGGATCCGCGGCCGCTTACAACCGGCTGCGTTTGGC |
| **UL69** | CCGGAATTCTCTCGAGGTGAGCTGCACTCACGCGGCCGT | CGCGGATCCGCGGCCGCTTAGTCATCCATATCATCGCTGTAAC |
| **UL71** | CCGGAATTCTTGTCGACCAGCAGAATCATACTCTGTTGCGA | CGCGGATCCGGTACCTCACACGGAGGACAGCAAGG |
| **UL72** | CCGGAATTCTCTCGAGGTCTTACGATGTTTACCGATCGAAT | CGCGGATCCGCGGCCGCTCAGGGATGACGGGGAGGTTT |
| **UL73** | CCGGAATTCTCTCGAGGTGAGTGGAACACACTAGTACTAG | CGCGGATCCGCGGCCGCTCAATAGCCTTTGGTGGTGGT |
| **UL74** | CGCGGATCCTGTCGACCGGGAGAAAGGGAGAGATGAGA | CGCGGATCCGGTACCTTACTGCAACCACCACCAAAGG |
| **UL75** | CCGGAATTCTTGTCGACCCGACCAGGCCTCCCCTCCTA | CCGGAATTCGGTACCTCAGCATGTCTTGAGCATGCGGT |
| **UL77** | GGGAATTCCATATGAAGATCTCTAGTCTGTTGCACACCTTTTGGC | CCGGAATTCGGTACCTTACAACACCGCCACGCTCGGA |
| **UL79** | CCGGAATTCTCTCGAGGTATGGCCCGCGACGAAGAGAA | CGCGGATCCGCGGCCGCTCACACGTCGTTAGCCAGCGT |
| **UL80** | GGGAATTCCATATGAAGATCTCTACGATGGACGAGCAGCAGC | CCGGAATTCGGTACCTTACTCGAGCTTATTGAGCGCA |
| **UL82** | CCGGAATTCTTGGCCATGGAGGCCTCTCAGGCATCGTCCTCGCC | CGCGGATCCCTCGAGCTAGATGCGGGGTCGACTGC |
| **UL83** | CCGGAATTCTTGTCGACCGAGTCGCGCGGTCGCCGTTGT | CGCGGATCCGGTACCTCAACCTCGGTGCTTTTTGGGCGT |
| **UL84** | GGGAATTCCATATGAAGATCTCTCCACGCGCCGACCCCAACC | CCGGAATTCGGTACCTTAGAGATCGCCGCAGACCATG |
| **UL85** | CCGGAATTCTTGTCGACCGCGGCCATGGAGGCCAACAT | CGCGGATCCGGTACCTCAGCCTTTAAATATGCAGGTC |
| **UL86** | GGGAATTCCATATGAAGATCTCTGAGAACTGGTCGGCGCTCGAG | CCGGAATTCGGTACCTCACGAGTTAAATAACATGGATTGC |
| **UL88** | GGGAATTCCATATGTTGTCGACCATGGAAGCCGCGGCCGCTG | CCGGAATTCCTCGAGCTAGGCACGCAGCAGAGCCA |
| **UL89.1** | CCGGAATTCTTGTCGACCTTGCGCGGAGACTCGGCC | CGCGGATCCGGTACCTTAGTTGGTGTTGTAGCAACTGG |
| **UL89.2** | CCGGAATTCTCTCGAGGTAGCATCCGAGGACAAAACTTC | CGCGGATCCGCGGCCGCCTAGCTGACCCTGAAACGGA |
| **UL93** | GGGAATTCCATATGTTGTCGACCGAAACGCACCTGTATTCGGATC | CGCGGATCCGGTACCCTAAAGATCGTCGAACGGCAAG |
| **UL94** | CCGGAATTCTCTCGAGGTGCTTGGCGCAGCGGGCTTTG | CGCGGATCCGCGGCCGCTTAGTGCACTAGGTTCTTAAGCAC |
| **UL96** | CCGGAATTCTCTCGAGGTACGTCGGTCAACAAACAGCTC | CGCGGATCCGCGGCCGCTTAGACGGCGTCGTCGACCT |
| **UL97** | GGGAATTCCATATGTTGTCGACCTCCTCCGCACTTCGGTCTCG | CCGGAATTCGGTACCTTACTCGGGGAACAGTTGGC |
| **UL99** | CCGGAATTCTCTCGAGGTGGTGCCGAACTCTGCAAACGA | CGCGGATCCGCGGCCGCTTAAAAGGGCAAGGAGGCGGC |
| **UL100** | CCGGAATTCTCTCGAGGTGCCCCCTCGCACGTGGATAA | CGCGGATCCGCGGCCGCTCAAGCGTCCTCGAAGTCTTCA |
| **UL103** | CCGGAATTCTTGTCGACCGAGGCCCTGATGATCCGCG | CGCGGATCCGGTACCTCACTCTTCCTCTCCCCGTTC |
| **UL104** | CCGGAATTCGCGGCCGCAGAGCGAAACCACTGGAACGAA | CGCGGATCCGCGGCCGCCTAGTGAAATCCGTATGGACCT |
| **UL112.1** | GGGAATTCCATATGTTGTCGACCGATCTGCCTACTACCGTCGT | CCGGAATTCGGTACCTTAGTCGTTCTCGGAGGAGGGA |
| **UL112.2** | GGGAATTCCATATGAAGATCTCTGGCTCTCCTCCCCTCCGG | CGCGGATCCGGTACCTTAATCGTCGAAAAACGCCGC |
| **UL115** | GGGAATTCCATATGTTGTCGACCTGCCGCCGCCCGGATTGCG | CGCGGATCCGGTACCTTAGCGAGCATCCACTGCTTGA |
| **UL119** | CCGGAATTCTTGTCGACCTGTCCCGTACTGGCGATCG | CGCGGATCCGGTACCTTACTTACCTTTATCACCGGG |
| **UL122** | CCGGAATTCTTGTCGACCCTGCCCCTCATCAAACAGGAA | CGCGGATCCGGTACCTTACTGAGACTTGTTCCTCAG |
| **UL132** | CCGGAATTCTTGTCGACCCCAGCACCGCGGGGTCTCCTT | CGCGGATCCGGTACCCTAGTCGTACTCGGGATCTCTGA |
| **US22** | CCGGAATTCTTGTCGACCTCCCTACTCACCAAAGCCGC | CGCGGATCCCTCGAGTTAGGGACCCGGGTCTGGTC |
| **US23** | GGGAATTCCATATGTCTCGAGGTTGGCGTACACGTTGGGAAGACG | CGCGGATCCGCGGCCGCCTACACAAAGTGCTCCCGAAAATC |
| **US24** | GGGAATTCCATATGTCTCGAGGTATGGATCCGGCTGCGGGTTCT | CCGGAATTCGCGGCCGCTCAAATCTGGATGTACTCGCGCA |
| **US27** | CCGGAATTCTTGTCGACCACTACATCTACTACAACTACCAC | CGCGGATCCGGTACCTTACAACAGAAATTCCTCCTCC |
| **RL10** | CCGGAATTCTTGTCGACCTATCCGCGTGTAATGCACG | CGCGGATCCGGTACCTCAGACGTCGTCGTCCTC |
