# Supplementary material for: Yeast Two Hybrid Analyses Reveal Novel Binary Interactions between Human Cytomegalovirus-Encoded Virion Proteins
Source: PLoS One. 2011 Apr 1;6(4):e17796. doi: 10.1371/journal.pone.0017796 (PMC3069980; doi:10.1371/journal.pone.0017796)
Supplement: Table S2 — Yeast two-hybrid mating results between constructs that contained DNA sequences coding for 59 binding domain (BD) and 59 activation domain (AD) fusion proteins. “AA” represents BD fusion proteins that are identified as autoactivators. “−” represents matings that resulted in no interactions. Highlighted “P” represents positive interactions by YTH mating. There was no significant difference in the growth of the diploid yeast cells representing all the positive interactions. (DOC) [file pone.0017796.s002.doc]

**SUPPORTING INFORMATION**

**Table S2.** Yeast two-hybrid mating results between constructs that contained DNA sequences coding for 59 binding domain (BD) and 59 activation domain (AD) fusion proteins. “AA” represents BD fusion proteins that are identified as autoactivators. “-“ represents matings that resulted in no interactions. Highlighted “P” represents positive interactions by YTH mating. There was no significant difference in the growth of the diploid yeast cells representing all the positive interactions.

CP-Capsid Protein; TP-Tegument Protein; EP-Envelope Protein
